# Individual differences in social homeostasis

**DOI:** 10.3389/fnbeh.2023.1068609

**Published:** 2023-03-10

**Authors:** Karen L. Bales, Sally Hang, John P. Paulus, Elaina Jahanfard, Claudia Manca, Geneva Jost, Chase Boyer, Rose Bern, Daniella Yerumyan, Sophia Rogers, Sabrina L. Mederos

**Affiliations:** ^1^Department of Psychology, University of California, Davis, >Davis, CA, United States; ^2^Graduate Group in Psychology, University of California, Davis, Davis, CA, United States; ^3^Graduate Group in Neuroscience, University of California, Davis, Davis, CA, United States; ^4^Graduate Group in Human Development, University of California, Davis, Davis, CA, United States; ^5^Graduate Group in Animal Behavior, University of California, Davis, Davis, CA, United States

**Keywords:** social homeostasis, stress reactivity, biobehavioral stress tendencies, attachment, sex differences, personality, culture, epigenetics

## Abstract

The concept of “social homeostasis”, introduced by Matthews and Tye in 2019, has provided a framework with which to consider our changing individual needs for social interaction, and the neurobiology underlying this system. This model was conceived as including detector systems, a control center with a setpoint, and effectors which allow us to seek out or avoid additional social contact. In this article, we review and theorize about the many different factors that might contribute to the setpoint of a person or animal, including individual, social, cultural, and other environmental factors. We conclude with a consideration of the empirical challenges of this exciting new model.

## 1. Introduction

**Figure 1:**
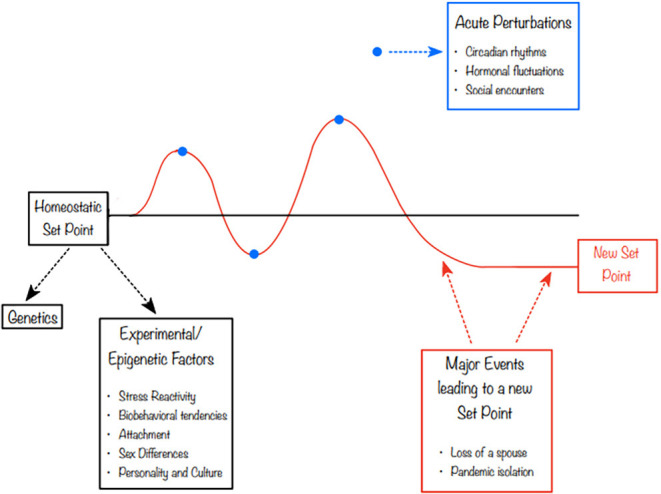
Social utility at any given time point is never static. In this figure, we highlight the role that different experiences, including epigenetic processes and environmental factors, can play in both initial setpoint and changes over time. Both acute and chronic social and stress-related processes can result in short- or long-term adjustment to the setpoint.

In 2019, Matthews and Tye (2019) proposed a model of “social homeostasis”. The literature on the neurobiology of social behavior has struggled to reconcile situations in which, for instance, a “prosocial” hormone like oxytocin (OXT) was elevated during periods of social challenge such as grief or separation (Taylor et al., 2000; Bales and Rogers, 2022). In contrast, recent stress literature conceptualized the stress axis as a homeostatic system with set points and a process of returning to those set points (McEwen, 2017). Calling this process “allostasis”, the cumulative effect of allostasis was termed “allostatic load.” Matthews and Tye (2019) proposed that social contact, like other systems which receive external input that has effects on neurobiological and physiological processes, could be conceptualized as having a detector (sensory system), a control center (compares the input to homeostatic set point) and an effector that drives a response. The feedback response would then be intended to nudge the system back to its setpoint, although extreme acute or chronic exposures could result in a new set point (Figure 1). This reconceptualization of social processes as social homeostasis or social allostasis would allow scientists to view, for instance, the increased OXT sometimes displayed by a grieving individual as a compensatory response in an allostatic attempt to return to a homeostatic set point. OXT may compensate for social loss similarly to the way that in stress biology, cortisol may rise in response to a psychosocial or metabolic challenge.

In a follow-up article, Lee et al. (2021) outlined some of the mechanisms underlying the processes of social homeostasis and applied the model to the example of social isolation. In their words, the control center would have to recognize “the perceived social environment—which is a high-dimensional state (including factors such as group size, relative rank, hierarchical structure, individual pairwise relationships, and observational learning), where subjective factors including previous experience and preference are paramount.” We were fascinated by this idea of social homeostasis and the potential of individuals to respond differently based on the many factors that lead to perceived social environment. Rather than focusing on the response of the system to a specific social challenge, we chose to ask the question, what factors interact with and predict the functioning of this system, particularly the set point of the control center? How do genetic and epigenetic factors, as well as other sources of individual differences and experiences, predict what level of social interaction serves as the setpoint, or predict the flexibility of the system as a whole? Broadly, what factors drive individual differences in the social homeostatic system? In this article, we review the evidence for the effects of stress reactivity, biobehavioral tendencies, attachment, sex differences, neurodiversity, and personality as potential moderators of individual differences in social homeostasis. We then discuss the role of epigenetic processes and methodological complications in studying social homeostasis.

## 2. Social homeostasis and stress reactivity

The social homeostatic model describes the neurobiological mechanisms of registering, regulating, and responding to the need for social contact. This ability to acknowledge and adapt to significant changes in social connection is deeply intertwined with the stress response system. Research in animal models and humans has demonstrated that a deficit or surplus in social contact can elicit a stress response (see Lee et al., 2021 for examples). The extent to which this stress response facilitates or interferes with social connectedness and affects efforts to maintain social homeostasis depends on the context of the stressor and the sensitivity of the stress response system. This section focuses on the latter, highlighting how individual differences in stress reactivity can pose risks or support social homeostatic resilience in the face of acute and chronic social-ecological changes.

Stress reactivity is best conceptualized as biological sensitivity to context. This definition, initially proposed by Boyce and Ellis (2005), argues that an exaggerated or attenuated stress response can be an evolutionary adaptation to the demands of the environment. The sensitivity of the stress response system is structured and calibrated *via* complex and continuous interactions between genetics and life experiences, particularly early caregiving interaction (Gunnar and Donzella, 2002; Boyce and Ellis, 2005; Flinn et al., 2011). Wide-ranging variations in genetic and environmental conditions can lead to individual differences in stress reactivity which have been documented in both animals, primarily rodents and primates (see Meaney, 2001 for review) and in humans (Cacioppo et al., 1998; Alkon et al., 2003). However, the contributions of specific genetic polymorphisms to the sensitivity of the stress response system remain controversial (Fergusson et al., 2011). Nonetheless, profiles of stress reactivity have been captured in the neurobiology and behaviors of human and animal models with a significant focus on the patterns of neuroendocrine indicators of hypothalamic-pituitary-adrenal (HPA) axis activity, namely cortisol (Gunnar et al., 2009; Foley and Kirschbaum, 2010).

*Hyper*cortisolism and *hypo*cortisolism are among the hypothesized effects of early life adversity in which the HPA axis response to a stressor becomes exaggerated or attenuated, respectively (Davies et al., 2007). Studies in humans have proposed that stress reactivity demonstrated by changes in cortisol may influence decision-making processes that inform social behaviors (see Starcke and Brand, 2012 for review). Furthermore, stress reactivity is developmentally acquired and solidified over time, paralleling maturational declines in plasticity and resulting in relatively stable phenotypic presentations (Turkheimer and Gottesman, 1991; Davidson et al., 2000). While the stress response is a normative component of the social homeostatic model, there remains great potential for future research efforts to unpack the ways in which variations in HPA axis reactivity may influence social homeostatic set point and efforts to cope with significant fluctuations in social utility (social utility is defined by Lee et al., 2021, as the product of social quality and social quantity).

The buffering effect of social relationships on stress has been well documented in the literature (see Cohen and Wills, 1985 for review). More recent human research in this area has demonstrated that high-quality, supportive relationships can go so far as to buffer against the dysregulation of biological stress systems such as the HPA axis (Carroll et al., 2013; Brooks et al., 2014), and lessen the cortisol reaction to a social stressor (Hostinar and Gunnar, 2015). Thus, it is reasonable to postulate that stress reactivity may influence perceived need or desire for social input, in other words, social homeostatic set point. On the one hand, high stress reactivity may encourage greater social input to elicit the buffering effects of social relationships, increasing sensitivity to social utility changes. On the contrary, high stress reactivity might also increase perceptions of social threat and trigger maladaptive coping behaviors like social withdrawal, which could ultimately motivate set point adaptations in conditions of a chronic discrepancy between the set point and social utility. These proposed relationships between the difference in stress reactivity and the homeostatic regulation of social connection are just some of the many exciting avenues for future inquiry to demystify the dynamic interplay between these two systems and the adaptive or maladaptive functioning that results.

Compelling evidence supporting the influence of biological stress reactivity on social homeostatic set point comes from research in animal models that have been genetically manipulated to have blunted or enhanced neuroendocrine stress responses. Wistar-Kyoto rats with a genetically exaggerated stress response have been shown to exhibit higher levels of social withdrawal (Nam et al., 2014); reductions in social behavior in response to stressors have also been observed in rat strains with *hypo*-sensitive neuroendocrine stress reactivity (Pardon et al., 2002). Literature on loneliness in humans also partially illustrates and supports the idea that high stress individuals are relatively more sensitive to changes in social utility as self-reported perceived stress has been linked with higher levels of self-reported loneliness and social stress sensitivity (Cacioppo and Hawkley, 2009; Nowland et al., 2018). The relationship between these perceived measures of stress reactivity and subjective experience of social threat and deficit have been linked—albeit inconsistently—to cortisol patterns, with many studies failing to find a direct association (Edwards et al., 2010; Schlotz et al., 2011; Nowland et al., 2018). Similar research on Social Anxiety Disorder (SAD) has shown associations between heightened perceived stress and social anxiety symptoms but did not find associations between SAD and cortisol levels (Nelemans et al., 2017). Both loneliness and SAD are directly linked to an individual’s affective, social experience, and appraisal of their set point. These findings suggest that perceived stress reactivity may indeed influence perceived social needs and therefore play a role in the calibration of the social homeostatic set point. Nonetheless, there remains a gap in our understanding of how biologically encoded stress reactivity may influence social homeostatic set point and social utility appraisal.

Research on the effects of stress reactivity on behavioral efforts to maintain social homeostasis is richer and more readily available. There is a growing body of literature that has demonstrated that acute stress can promote prosocial and affiliative behaviors in humans (Taylor, 2006; von Dawans et al., 2012), with some studies connecting changes in social behaviors post stressor with increases in cortisol levels (Smeets et al., 2009; Zhang et al., 2019). Conversely, studies of social isolation in multiple species of rodents, which is accompanied by hyperadrenocorticism, have found decreases in prosocial behavior and increases in antisocial behaviors, including aggression and depressive symptoms (see Beery and Kaufer, 2015 for a review). Human research on social withdrawal has also found high cortisol levels to be linked to social reticence (Schmidt et al., 1997) and behavioral inhibition (Spangler, 1998) in young children. These findings suggest that both high and low cortisol reactivity profiles can have disruptive effects on efforts to maintain social homeostasis if there is a social utility deficit. Furthermore, low cortisol reactivity has been linked to increased antisocial behaviors, aggression (McBurnett et al., 2000; Shirtcliff et al., 2005), and externalizing behaviors in children (Obradović et al., 2010). However, the effects of aggressive and externalizing behavior on perceived social connectedness have produced mixed, age-dependent effects. One study demonstrated that higher aggression in children was related to higher peer rejection and loneliness (Cassidy and Asher, 1992). A similar study examining the persistence of this relationship in adolescence did not find a significant link between aggression and loneliness (Parkhurst and Asher, 1992).

Research in both human and animal models to date has begun to unpack the many ways in which stress reactivity influences social behaviors, yet the extent and magnitude of these effects are significantly influenced by species, age, and context and are unlikely to be driven by cortisol reactivity profiles alone. As such, there is a wide opportunity for future research to explore how these two models inform each other. To further understand the influence of stress reactivity on the effector system of the social homeostatic model, we suggest an intersectional approach that combines developmental and ecological effects, with strong attention to an individual’s perception of their own stress reactivity. Nevertheless, the current canon of research supports Boyce and Ellis (2005) in their argument that stress reactivity is an adaptive process that can both promote and impede an individual’s ability to meet their social homeostatic needs.

## 3. Biobehavioral stress tendencies: tend and befriend

As detailed in the previous section, the HPA axis responds to stressors by ramping up a hormone cascade that prepares the body to handle the challenge. Although both men and women experience these adaptive responses, there are reports of sex differences in coping strategies. For example, Taylor (2006) “tend-and-befriend” theory states that under stress, our ability to tend to our offspring or befriend others is an alternative to the fight-or-flight response. According to the tend-and-befriend theory, this affiliative stress response is carried out by neuropeptide concentration level changes, particularly in OXT (Taylor et al., 2000; Youssef et al., 2018). This theory proposes that the perceived gaps in social relationships help elevate peptides such as OXT, which motivate the biobehavioral stress response to take actions to restore social connection. This hypothesis was based on the attachment-caregiving response, resulting in befriending behavioral tendencies as a part of the stress response. These tendencies may be particularly prevalent in females.

A central point of Taylor’s tend-and-befriend theory is that under stressful circumstances, females may be more likely to react with prosocial, affiliative behaviors compared to fight-or-flight tendencies. These pro-affiliative behaviors could be an adaptive response due to the unique type of stressors that women face (e.g., pregnancy and child-rearing). As stressors evolve, mechanisms for maintaining adequate levels of social connection would also evolve. These behavioral tendencies may be an adaptive coping tool used to understand the biobehavioral differences or similarities, between men and women throughout the lifespan as studies lacked women participants prior to 1995 (Taylor et al., 2000). In studying the tend-and-befriend model, research has found that acute stress increased prosocial behavior in men (von Dawans et al., 2012) and positive associations were seen in elevations of cortisol levels and altruistic decisions in men (Singer et al., 2017); but those studies did not include women. One study that did investigate the role of sex in social decision-making tasks found that men with higher cortisol levels were more generous, particularly for men with lower empathic concern (Zhang et al., 2019). Studies that included women have found that women in the “stress” group tended to engage in more trustworthiness and affiliative behaviors (von Dawans et al., 2012), however, the relationship with cortisol reactivity was not studied.

OXT also plays a role in attention reorienting in social situations. During stress, OXT modulates the salience of social cues through its interaction with the dopaminergic system (Shamay-Tsoory and Abu-Akel, 2016; also see Groppe et al., 2013 and Love, 2014), in studies using rats (Shahrokh et al., 2010) and human participants (Loup et al., 1991; Rilling et al., 2012; Scheele et al., 2013). OXT has also been shown to dampen the fear circuitry in the central nervous system (as reported in Delgado, 2008). When social connections dip below an adequate level, the social neurocircuitry would act as a mechanism to increase OXT levels to motivate social behaviors to meet an adequate level of social connection (humans: Taylor et al., 2000). Measuring empathic concern and cortisol levels could also be utilized as a mechanism to distinguish between these biobehavioral tendencies in men and women (Zhang et al., 2019). Experimental designs could further refine methods to include ecological testing along with pre and post stressor assessments to assess how biobehavioral tendencies relate to an individual’s social homeostatic set point. Additional considerations include testing in varying populations (e.g., developmental to adults), cultural contexts, and diverse non-WEIRD communities. Further research is needed to understand the role that differing adaptive biobehavioral responses during stressful social conditions play in maintaining a social homeostatic set point.

## 4. Attachment

The attachment system may also play a salient role in the maintenance of social homeostasis, as it establishes its own baseline sense of internal security tied to the proximity of an attachment figure (Bowlby, 1969). An attachment system may function as a parallel homeostatic system that works in tandem with social homeostasis to govern an individual’s social behaviors. Attachment theory posits that individuals generally form either an insecure attachment style (i.e., anxious-insecure attachment, avoidant-insecure attachment) or a secure attachment style (Hazan and Shaver, 1987). However, it is crucial to note that some individuals may exhibit multiple flavors of distinct attachment dimensions, resulting in a disorganized attachment style (Bakermans-Kranenburg et al., 2003).

Attachment styles are generally rooted in early caregiving experiences but may shift across a lifespan (Fraley et al., 2021). In adulthood, our attachment figures tend to be the romantic partners with whom we form relationships, or pair bonds. Given that attachment bonds exist across species, pair bonds formed by some socially monogamous animals (i.e., animals who form long-term pair bonds with a single mate, such as prairie voles, California mice, and titi monkeys) provide critical insight into the neurochemical mechanisms underlying these complex dyadic processes (Bales et al., 2021). Prior research on socially monogamous prairie voles suggests that key hormones and neurotransmitters are involved in pair bonding (i.e., formation of an adult attachment), including OXT, dopamine (DA), and arginine vasopressin (AVP; Wang and Aragona, 2004). It is plausible that attachment and social homeostatic systems are communicating with each other, thus potentially releasing similar neuropeptides during social homeostatic processes.

Like social homeostatic systems, attachment systems are sensitive to external social factors, such as loss of attachment figure proximity. When the attachment system is perturbed, an individual will deviate from their setpoint (i.e., secure attachment, anxious-insecure attachment, or avoidant-insecure attachment). An individual with attachment anxiety may engage in hyperactivating strategies to increase proximity to the attachment figure (Mikulincer et al., 2003; Berant et al., 2005). Hyperactivating strategies (e.g., pleading, demanding, calling) aim to coerce an unavailable attachment figure to re-engage with a relationship, which may lead to overdependence on a partner (Shaver and Hazan, 1987). An activated attachment system can only revert to its baseline state when the attachment figure reinforces a sense of security by sufficiently responding (Berant et al., 2005). Given that hyperactivating strategies compel individuals with attachment anxiety to intensely seek out their attachment figures, their social homeostatic setpoints will likely shift in tandem with the proximity of these attachment figures. Conversely, an individual with insecure attachment avoidance may engage in deactivating strategies to maximize distance from the attachment figure to mitigate pain associated with their absence (Mikulincer et al., 2003; Berant et al., 2005). These deactivating strategies may lead to “compulsive self-reliance”, in which an individual intentionally evades social environments that may foster greater intimacy (Berant et al., 2005). Consequently, these kinds of regulatory strategies may directly impact the social homeostatic set point by reducing social support and engagement. Furthermore, a wealth of prior research suggests that individuals with high levels of secure attachment are more likely to seek out social support than insecurely attached individuals, especially during distressing periods (Ognibene and Collins, 1998; DeFronzo et al., 2001). Additionally, securely attached individuals are more likely to report they have cultivated dependable social networks that sufficiently provide support (Wallace and Vaux, 1993). Thus, this body of research affirms that the attachment system substantially impacts the level of social contact one may seek with others (i.e., social homeostatic setpoint). Therefore, future research could investigate whether the attachment system reliably predicts social homeostatic patterns.

The attachment framework may offer further insight into how the social homeostatic set point may differ from individual to individual, and how it may evolve across a lifespan. Prior research suggests that while adult attachment is linked to early caregiving experiences, these early experiences do not always determine adult attachment outcomes (Fraley and Roisman, 2019). Fraley et al. (2021) found that while individuals often returned to their baseline attachment style after significant life events, people also reported an enduring shift in attachment style. Individuals shifted from either an insecure style to a secure style or *vice versa* after approximately a quarter of all studied events, such as the birth of a child, marriage, or illness (Fraley et al., 2021). Furthermore, prior research also suggests that the mental construal or interpretation of either positive or negative events may substantially influence whether attachment changes endure (Davila and Sargent, 2003; Fraley et al., 2021). Considering that a significant population has reported durable attachment shifts in adulthood, future longitudinal research could investigate whether social homeostatic set points may operate similarly.

An additional focal point of interest may be rooted in a dyadic attachment perspective, which posits a relationship may form a cohesive, interactive dyadic attachment system that co-regulates romantic partners. Timmons et al. (2015) demonstrated that romantic dyads may coregulate their blood pressure, cortisol, respiration, among myriad other physiological processes. These findings may suggest that dyadic co-regulation and linkage within adult pair bonds could produce a unique dyadic physiological and psychosocial system, including a dyadic social homeostatic set point and attachment system. While dyadic attachment is still an emerging concept within the field, studies have found that dyadic co-regulation processes may contribute to relationship wellbeing, because an individual’s secure attachment may mitigate the negative effects of a partner’s insecure attachment (Banse, 2004; Overall and Simpson, 2015). A dyadic homeostatic system may have significant implications for health across the lifespan, given that romantic relationships and social networks are robustly linked to health outcomes (Cacioppo and Hawkley, 2003; Smith and Christakis, 2008; August et al., 2016).

## 5. Sex differences

In the previous sections, we argued that sex differences might impact an individual’s set point as well as behavioral effector mechanisms, particularly in the context of stress. The two main factors in the development of stress-related disorders, like depression, are sex and social context (Karamihalev et al., 2020). In humans, cortisol level responses can be influenced by sex. One study found that men had significantly greater cortisol responses to achievement challenges, while women showed greater cortisol responses to social rejection challenges (Stroud et al., 2002). Based on social context, being more resilient to rejection challenges or having a higher ability to cope with stress could affect an individual’s set point. Women have also been found to be more resilient to stress-induced attention deficits, but more vulnerable to stress-induced hyperarousal (Bangasser et al., 2019).

For women and female animals, the menstrual or estrous cycle should also be considered when discussing variations in set point. The female brain has mechanisms that modulate the monthly fluctuations of sex steroids throughout the menstrual cycle (Donner and Lowry, 2013). These ovarian hormones can influence cognitive, stress-related, and emotional outcomes (Ney et al., 2019). The subjective experience of fear and panic in response to a social stress test has been found to be greater in women (Kelly et al., 2008). Women with PTSD report higher depression and phobic anxiety symptoms during the early follicular phase of their menstrual cycle (Ney et al., 2019).

An individual set point can be greatly shifted by stress-induced disorders, such as depression, which also show sex differences. Social defeat, an experimental paradigm in which one animal is defeated by another, aggressive animal, is a useful way to study stress responsiveness in animals and is a model for human depression (Page et al., 2016); it also induces marked sex differences in behavioral response (Steinman and Trainor, 2017). The effects of social defeat in California mice (*Peromyscus californicus*) are independent of adult gonadal steroids and are instead associated with sex-specific changes in AVP and OXT (Steinman and Trainor, 2017). The sex steroids are removed by gonadectomy, so sex differences must be due either to developmental hormonal or sex chromosome complement effects (Seney and Sibille, 2014). In California mice, after exposure to social defeat, males exhibited increased aggression and reduced cognitive flexibility while females showed social avoidance and low aggression (Steinman and Trainor, 2017). In Syrian hamsters (*Mesocricetus auratus*), a “single social defeat in puberty increases susceptibility to later social defeat in both males and females” (Rosenhauer et al., 2017). This early exposure to social defeat may directly shift a set point, which would then allow future social defeats to affect the individual more drastically and easily. This animal paradigm has clear translational relevance for humans undergoing social stressors in particular, and thus once again suggests that sex differences may interact with social stressors to affect social homeostasis.

## 6. Personality and culture

Personality (humans)/temperament (animals), and the nexus between personality and cultures may play a key role in determining one’s social set point and the flexibility of the social homeostatic system. Cultures have been long dichotomized into either collectivist or individualistic, the former associated with Eastern societies and the latter associated with Anglo-Western societies. Furthermore, membership in one or the other also comes with distinct values, goals, and motivations that are encouraged (Markus and Kitayama, 1992; Schwartz, 1994).

Extraversion is understood to be one of the overarching personality traits in many personality taxonomies, including the canonical Five-Factor Model/Big Five Inventory, that subsumes other characteristics, chiefly sociability and gregariousness (Schmitt et al., 2007). While extraversion has been well established as a cultural universal (Lucas et al., 2000; Bond et al., 2020), in their review article investigating the geographic distribution of personality profiles, Schmitt et al. (2007) found a significant main effect of a nation on extraversion. Namely, East Asia, South America, and South/Southeast Asia scored significantly lower on measures of Extraversion than the seven other regions tested. These lesser extraverted regions though are also overall less likely to seek social support to cope with stressors than their European American counterparts (Kim et al., 2008). These cross-cultural variations may affect social set points at the individual level. Personality may also interact with potential challenges in one’s life that may disrupt their set point. For instance, a negative association was found between extraversion and PTSD, and extraversion and bereavement (Jakšić et al., 2012; Asselmann and Specht, 2020).

While human axes of personality, such as the Five Factor Model, have been thoroughly investigated and validated, there are few studies assessing the degree to which these axes can be mapped onto animal models. Gartland et al. (2022), in reviewing personality studies in animals, posit the trait sociability to be akin to our understanding of extraversion as a personality trait in humans, such that sociability reflects “an individual’s tendency or propensity to associate with other individuals, where the association is not driven by reproduction or aggression”. Animal temperament is also deemed to be generally stable throughout development (Réale et al., 2007). Moreover, across a multitude of animal taxa including wild great tits (Aplin et al., 2015), three-spined sticklebacks (Bevan et al., 2018), vervet monkeys (Blaszczyk, 2017), cockroaches (Planas et al., 2015), and even unicellular eukaryotes (Vogel et al., 2015), there are consistent individual differences in sociality.

Scientists have also identified genetic correlates of animal sociability. For example, Parker et al. (2019) found that varying levels of AVP in rhesus monkeys were associated with group differences in sociality. These findings and studies on dogs (Kis et al., 2014), mice (Sala et al., 2013), and chimpanzees (Staes et al., 2015) further inform our understanding of the ways in which differential social set points are- in part- determined by personality traits, in particular the trait sociability. Additional comparative research could endeavor to relate the frameworks described here (stress reactivity, attachment, and personality) in the theoretical framework of social homeostasis for each individual species as well as across species.

## 7. Genetic and epigenetic influences

The process of social homeostasis is influenced by situational components (such as previously mentioned environmental context and experience) and biological components (such as neuroendocrine factors; Fox and Calkins, 2003; Bell and Deater-Deckard, 2007). These individual differences arise from genetic and epigenetic effects producing differences in the self-regulatory process of social homeostasis. Lee et al. (2021) acknowledge that individual differences both across and within species with respect to patterns of social engagement contain a strong influence of genetic factors (Lim et al., 2004; Hoekstra and Coyne, 2007; Wang et al., 2008; Forkosh et al., 2019; Lee et al., 2021). Neural systems are influenced and regulated by genetics and epigenetic processes thus producing changes in many domains but namely in mood, behavior, and perception; all things relevant to the social homeostasis model (Houston et al., 2013). Here, we examine key points in the model and present the evidence for genetic and epigenetic effects on homeostatic set point and on effector systems, i.e., the responses to perturbation away from the set point.

Neurodivergent development provides a window into the role that genetics plays in this system. Autism spectrum disorder, or ASD (as well as neurodivergence overall) is a complex phenotype caused by genetic mutations in common regulatory pathways resulting in similar behavioral phenotypes (Persico and Napolioni, 2013). For neurodivergent people, social challenges to the set point would likely vary in magnitude just as they would with neurotypical individuals. However, neurodivergent people may face extra sensitivity to changes in their perceived social environment and have a harder time navigating the world through social interaction (Morewood et al., 2011; Morgan, 2019; Crompton et al., 2020). These challenges may be reflected in the functioning of the social homeostatic system.

Differences in the functioning of social homeostasis in neurotypical people and neurodivergent people could possibly be mediated by different functioning of the OXT and AVP systems. Some studies have found that people with ASD have altered levels of OXT (Gainer et al., 1995; Green et al., 2001), while others have found no relationship or altered correlations to symptoms (Rutigliano et al., 2016). OXT levels are also lower in people with Fragile X Syndrome (FXS; Hampson et al., 2011) and people with Prader-Willi Syndrome (PWS; Swaab et al., 1995; Oztan et al., 2022). On the other hand, people with Williams Syndrome (WS) may express higher levels of OXT than what is considered to be average for a neurotypical person (Henrichsen et al., 2011; Dai et al., 2012). AVP is a promising biomarker and potential therapeutic in neurodivergent people. Studies suggest that AVP is lower in people with ASD, FXS, and PWS (Gürkan and Hagerman, 2012; Johnson et al., 2016; Oztan et al., 2018; Wilczyński et al., 2019). Supplementation with AVP led to increases in social responsiveness in children with ASD (Parker et al., 2019). Due to the differences in neurotransmitter composition, it is important to note that social homeostasis at all levels—detector, control systems, and effectors—may function differently in neurodivergent people. The inclusion of these populations is therefore critically important to understanding individual differences in social homeostasis.

Both genetic and epigenetic factors may contribute to mental health conditions that can affect sociality and therefore a preferred level of social connectedness (set point). Growing evidence suggests that epigenetic regulation may underlie both the development of mood and stress-related disorders and the effectiveness of long-term clinical treatments (Abdolmaleky et al., 2005; Berton and Nestler, 2006; Sun et al., 2013; Fass et al., 2014; Nagy et al., 2018). One review found that transporter gene expression, genes encoding receptor systems, HPA axis factors, neurotrophic factors, and inflammatory factors affecting the brain are implicated in the risk of developing various mood disorders (Archer et al., 2013). Many neuropsychiatric drugs targeted to treat mood disorders have direct effects on epigenetic mechanisms such as histone deacetylases (Phiel et al., 2001) and histone demethylases (Lee et al., 2006). A study examining post-mortem brain tissue from patients suffering from mood disorders revealed that some epigenetic modifications (specifically different histone deacetylases that allow DNA to wrap more tightly around histones, affecting gene expression) are distinctly different from healthy control subjects, thus suggesting that epigenetic mechanisms play a fundamental role in the pathophysiology of such disorders (Hobara et al., 2010).

Temperament and behavioral traits likely develop early in life, through Gene x Environment interactions (i.e., epigenetic mechanisms), including those related to cooperativeness and likely other parameters relating to sociability (Svrakic and Cloninger, 2010). Some epigenetic modifications, such as DNA methylation, persist across long periods of time and some are reversible if prompted by specific environmental circumstances (Meloni, 2014). Therefore, epigenetic mechanisms can have profound effects on the social homeostatic set point of individuals both acutely and chronically. Many studies have found links between epigenetic effects and variation in personality traits (Kaminsky et al., 2008; Verhulst et al., 2016; Haas et al., 2018), though authors state that this area of research requires much more exploration.

Effector systems are described as coordinated behavioral and physiological responses that aim to restore oneself to a preferred baseline; in the case of social homeostasis, the level of preferred social engagement (Lee et al., 2021). As defined in that article, overall detected social interaction, or “social utility” is a function of both quantity and quality. A deviation from the preferred baseline for any homeostatic process, including social homeostasis, can be described as a type of stress. Resiliency has been described as an individual’s ability to adapt to acute stress as well as more chronic forms of stress (Feder et al., 2009). Feder et al. (2009) highlight interactions between an individual’s genetics and their exposure to environmental stressors and how that determines the adaptability of neurochemical stress response systems to new adverse experiences. This is relevant to social homeostasis as deviation from a preferred set point can potentially be an adverse experience, and various stress response systems play a key role in either promoting re-establishment of set point (acute) or the creation of a new one (chronic).

Individual differences in systems such as the HPA axis originate at the genetic level. Genes for mineralocorticoid and glucocorticoid receptors (GR) in the brain that are involved in setting the threshold and termination of the HPA axis response to stress through a feedback loop have been found to have functional variants in humans, thus creating variation in these systems (Feder et al., 2009). One example of this is that when undergoing a social stress test, carriers of a variant GR gene had higher cortisol responses (DeRijk and de Kloet, 2008). Another example is the association between genetic variation in a gene that codes for a protein that regulates GR sensitivity and inefficient recovery of HPA axis activity after exposure to the same social stress test previously mentioned (Ising et al., 2008). This identifies a potential risk factor for chronically elevated cortisol levels and in the context of social homeostasis may inhibit successful recovery of set point or even promote the establishment of a new set point.

Epigenetics provides the link between environment and internal adaptations as several epigenetic mechanisms (DNA methylation, histone modification, etc.) change genome function under exogenic influence (Berger et al., 2009). In the social homeostasis model, it is clear that epigenetic processes governing effector systems play a role in maintaining a preferred set point. An individual’s social utility wavers constantly, and variable upregulation and downregulation of relevant social drivers help maintain homeostasis. The final key point in the social homeostasis model where genetics and epigenetics play a crucial role in set point readjustment. Some processes affected by stress such as cellular apoptosis, neurogenesis, and chromatin modifications may be responsible for the long-term effects of stress (Lisowski et al., 2011). These lasting changes can be caused by changes in gene expression thus possibly modifying internal states such as a preferred set point (Stankiewicz et al., 2013). Epigenetics has been found to play a role in shaping stress-vulnerable phenotypes and promoting behavioral adaptations to chronic stress (Siegmund et al., 2007; Uchida et al., 2011).

## 8. Methodological challenges and approaches

The maintenance and adaptation of social homeostasis is a complex process informed by broad ecological and individual factors, as reviewed above (Figure 1). Understanding the influence of these factors on organisms’ social homeostatic set points and on the neurobiological mechanisms that maintain social homeostasis requires a novel methodology and advanced statistical approaches. In the following section, we outline the methodological challenges and potential approaches to studying individual differences in social homeostatic set points using human and animal models. We draw upon models akin to the social homeostasis model (e.g., Saxbe et al., 2020), other examinations of set points in psychosocial homeostatic processes (Capic et al., 2018) and best practices for reporting experimental animal models of social behavior as outlined by Matthews and Tye (2019).

The primary challenge in studying most homeostatic processes is that the set point at which homeostasis is maintained exists within a range that can be responsive to numerous factors that may vary within a short timeframe (Sterling, 2014). Social homeostatic set-point variability in humans can be informed by variability in mood (Bornas et al., 2015), physical space (Thiel et al., 2008), intergroup dynamics (Romero et al., 2009), and daily rhythm (Ehlers et al., 1988). Reliably measuring a set point requires intensive repeated measures data collection across different contexts of social engagement. In the study of well-being and happiness among humans, the set point has been established by collecting annual measurements across decades and deriving from them a trait-level measurement (Cummins et al., 2014). However, social homeostasis requires examination over multiple scales of time, from moment-to-moment to lifespan approaches. Ecological momentary assessments and other diary methods can examine the social homeostatic process across people’s daily lives where we can account for circadian rhythm, hormonal changes, fluctuations in social interaction, and mood variability.

Identifying important mechanisms in the social homeostatic process will require tightly controlled environments using experimental manipulation. The establishment of a set point and changes to that set point can be examined using time-lagged multilevel models that can parse out stable between-person differences and within-person deviations using person-mean centering (Bolger and Laurenceau, 2013; Hoffman, 2015). Similarly, state-space grids used in dynamic modeling can identify changes in the social stimuli and any subsequent effects on the social homeostatic process (Lewis et al., 1999; Saxbe et al., 2020). However, these methods are resource-intensive and cannot elucidate involved neural substrates.

Before applying the social homeostatic model to human research, refinement of animal models can help provide better insight into neurobiological mechanisms influencing social homeostatic set points. Attention should be given to the natural history and social structure of the species—making not just traditional rat and mouse models useful but also species with different social structures and different contexts of social rewards, such as prairie voles (Goodwin et al., 2019). Established paradigms in rodents that induce social isolation or overcrowding have the utility to be adapted in human samples (Mueller and de Wit, 2011). For example, the social place preference paradigm was originally developed using rodent models to assess social motivation but has been adapted to study social aversion among neurodivergent young children (Baron et al., 2020) and has been applied to human adults in a virtual reality setting (Childs et al., 2017). However, without rigorous and controlled animal experimental models using standardized procedures the validity of these paradigms becomes questionable and their application to humans loses utility (Matthews and Tye, 2019). To identify neural substrates of the social homeostatic process and set points requires intensive repeated sampling across ecological contexts and novel methodology that bears cross-species application.

## 9. Discussion

The concept of social homeostasis (Matthews and Tye, 2019; Lee et al., 2021) is potentially very useful in explaining the perceived needs for social interactions, as well as the underlying neurobiological and physiological mechanisms that promote the approach or avoidance of these interactions. In this article, we reviewed several areas of psychological research that lend themselves to explaining the homeostatic set point and the responsivity of the system. Common themes in the literature include interactions with stress, the role of experience and its encoding in epigenetics, and the need to consider sex differences, methodological issues, and the potential for important others to result in a dyadic set point.

## Author contributions

All authors contributed to the first draft of the article, reviewed, edited, and approved the final draft. All authors contributed to the article and approved the submitted version.
